# Central pancreatic body-preserving pancreatoduodenectomy for pancreatic head cancer following a combined total gastrectomy and distal pancreatectomy: a case report

**DOI:** 10.1186/s40792-019-0641-6

**Published:** 2019-05-22

**Authors:** Kazufumi Umemoto, Toru Nakamura, Toshimichi Asano, Takahiro Tsuchikawa, Keisuke Okamura, Takehiro Noji, Yoshitsugu Nakanishi, Kimitaka Tanaka, Satoshi Hirano

**Affiliations:** Department of Gastroenterological Surgery II, Division of Surgery, Hokkaido University Faculty of Medicine, North 15, West 7, Kita-ku, Sapporo, Hokkaido 060-8638 Japan

**Keywords:** Pancreas, Pancreatic cancer, Pancreatoduodenectomy, Indocyanine green fluorescence, Organ-preserving surgery

## Abstract

**Background:**

Following a total pancreatectomy and a total gastrectomy procedure, patients often present with nutritional and diabetes-related disorders, along with a corresponding decrease in their quality of life. Consequently, an appropriate surgical procedure should be selected, with which an R0 resection can be achieved while conserving optimum pancreatic function, in order to prevent adverse sequelae.

**Case presentation:**

We present a case of a 66-year-old female, with a history of primary gastric cancer, for which, she underwent total gastrectomy combined with a distal pancreatectomy, 21 years ago. She presented to us with a primary cancer of the head of the pancreas. We performed a central pancreatic body-preserving pancreatoduodenectomy, in order to conserve the pancreatic function. Since the splenic artery had already been resected in the earlier surgery, conserving the blood flow to the preserved pancreatic body was an important concern. By utilizing the techniques of preoperative angiography and intraoperative indocyanine green fluorescence imaging, the pancreatic body could be accurately resected while leaving its vascular supply intact and undamaged. Thus, the patient’s pancreatic endocrine function could be preserved.

**Conclusions:**

An accurate evaluation of the pancreatic vascular supply enabled the operating surgeon to perform a safe, central pancreatic body-preserving pancreatoduodenectomy, even in patients who have undergone a combined total gastrectomy along with a distal pancreatectomy.

## Background

While the occurrence of gastric cancer is showing a decreasing trend worldwide [1], it remains the third most common type of cancer in Japan [2]. Recent advances, in the techniques used for an early diagnosis, chemotherapeutic measures, and operative procedures, have improved the therapeutic outcomes for gastric cancer, which has correspondingly led to an increase in the number of patients who are able to achieve long-term survival [3]. This has resulted in more patients requiring surgery for pancreatic cancer after the gastric cancer surgery.

However, performing a pancreatic resection in a patient with a history of a total gastrectomy (TG) presents two problems. Firstly, losing the stomach and pancreas together can lead to a severe postoperative nutritional imbalance and a derangement of glucose metabolism [4]. Secondly, a post-TG pancreatic resection, particularly a pancreatoduodenectomy (PD), requires a high degree of skill, due to the presence of various anastomoses, anatomical complexities, localized adhesions, and other factors involved in the procedure [5]. A post-TG PD is reportedly associated with increased rates of postoperative complications, and operative mortality, indicating the formidable nature of the surgical procedure [5]. Ideally, a post-TG pancreatic resection should be performed safely and with an aim to preserve maximum organ function, while achieving a complete cure.

Here, we report our experience in performing a pancreatic body-preserving PD on a patient, who had previously undergone a TG combined with distal pancreatectomy (DP) and splenectomy and had now presented to us with a primary pancreatic head carcinoma. We were able to achieve a margin-negative resection by making an accurate preoperative determination of the pancreatic vascular supply preoperatively, using various diagnostic modalities.

## Case presentation

**Fig. 1:**
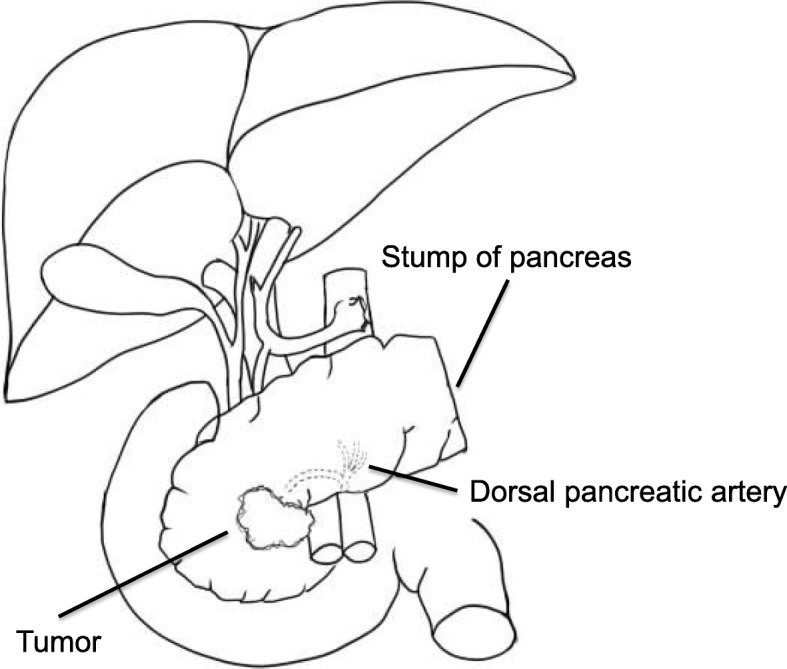
Preoperative abdominal schematic. A preoperative schematic indicating that a total gastrectomy, distal pancreatectomy, and splenectomy with Roux-en-Y reconstruction had already been performed in this patient

**Fig. 2:**
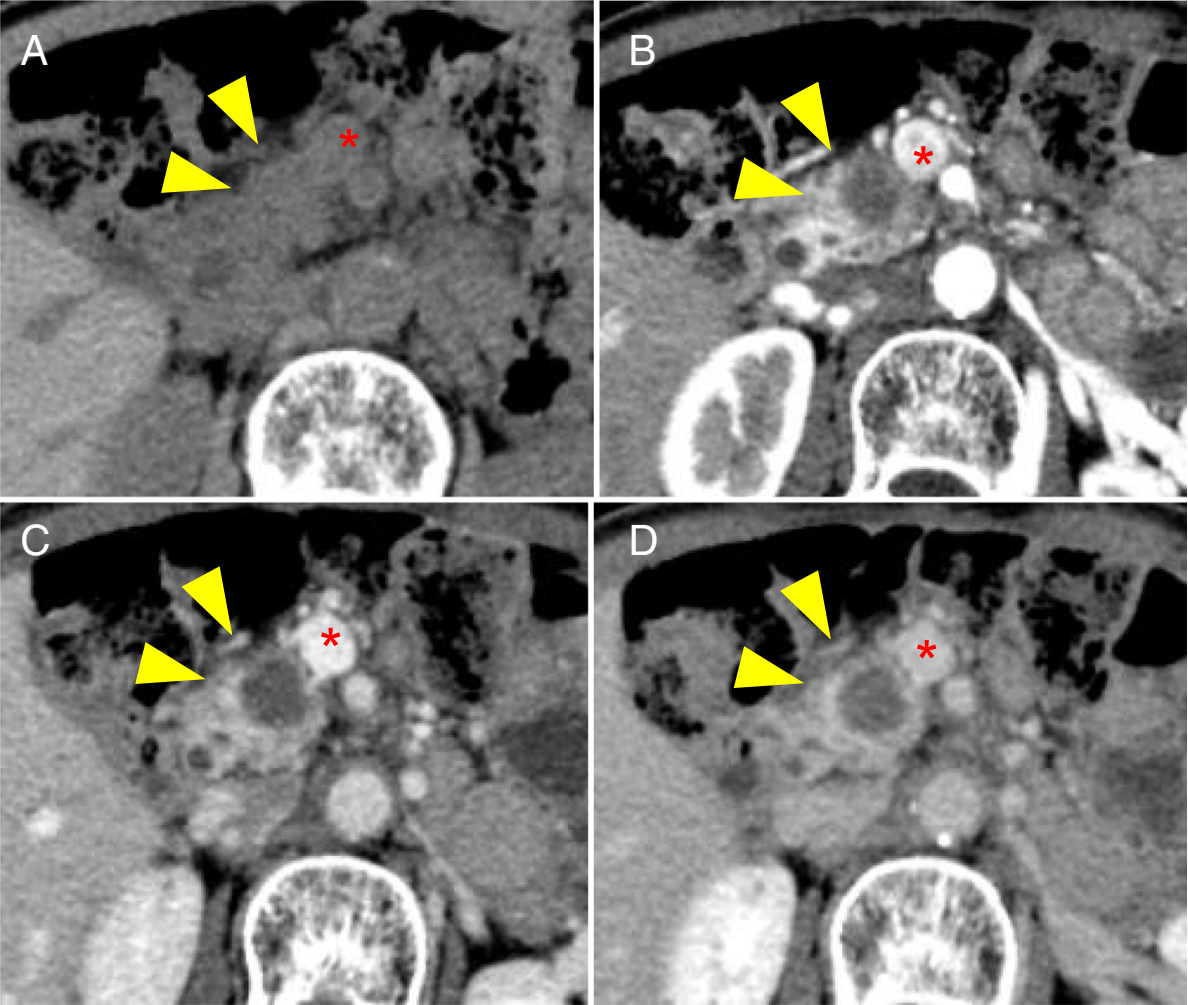
Preoperative abdominal computed tomography scan images. An enhanced abdominal computed tomography scan showing the **a** plane phase, **b** arterial phase, **c** portal phase, and **d** late phase images, performed after chemoradiotherapy, showing a 1.4 cm × 1.5 cm hypovascular mass with an irregular border in the head of pancreas (arrowheads), which has also infiltrated the superior mesenteric vein (asterisk)

The patient was a 66-year-old female referred to us for the management of pancreatic head carcinoma. At age 45, she had undergone a TG combined with a DP and a splenectomy for gastric cancer that had also involved the pancreatic tail (Fig. 1). A tumor on the pancreatic head was discovered during a follow-up for repeated adhesive intestinal obstructions. A computed tomography (CT) scan revealed a 15-mm hypovascular tumor on the head of the pancreas. It had an irregular border and was observed to be infiltrating the superior mesenteric vein (SMV). The patient’s CA 19–9 and CEA levels were found to be 24.0 U/ml (normal range, 0–37 U/ml) and 5.0 ng/ml (normal range, 1.0–6.5 ng/ml), respectively. Considering the patient’s post-TG status, a diagnostic endoscopic ultrasonography, or an endoscopic approach for a biopsy could not be attempted. Therefore, the diagnosis of a borderline resectable pancreatic cancer was made based on the National Comprehensive Cancer Network (NCCN) guidelines [6]. Neo-adjuvant chemotherapy with tegafur, gimeracil, and oteracil potassium (S-1) as well as radiotherapy (dosage, 50.4 Gy/28Fr) was attempted preoperatively. However, the treatment did not produce a marked change in tumor size (Fig. 2).

**Fig. 3:**
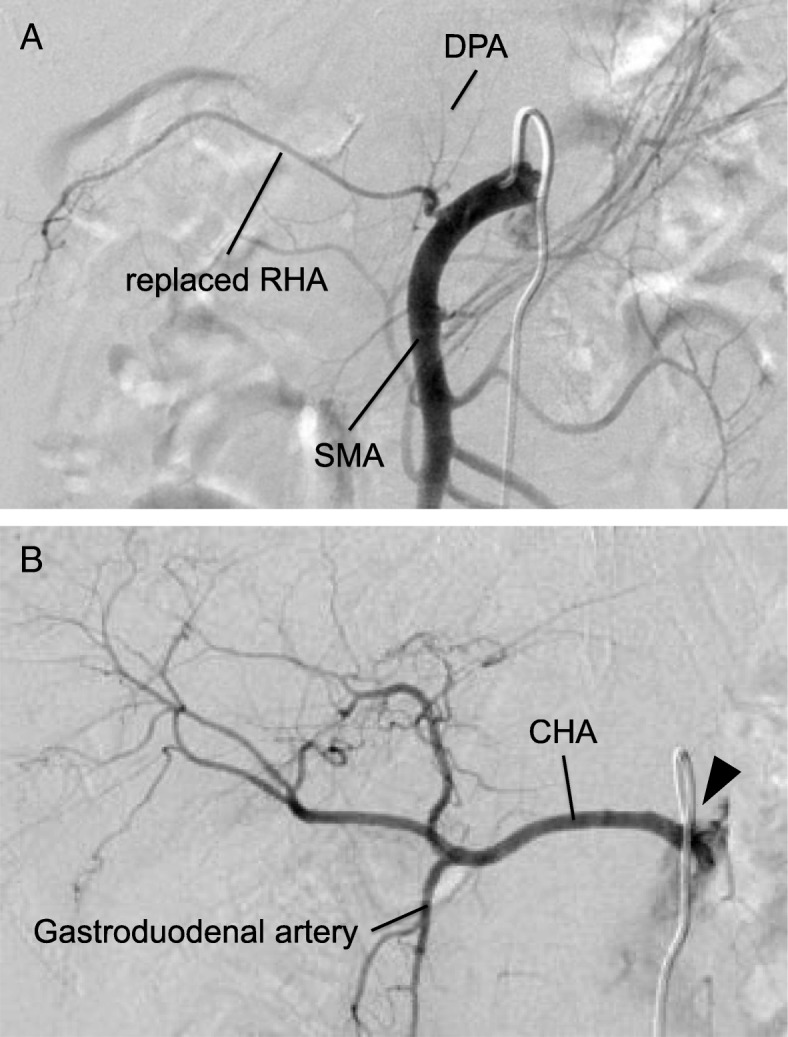
Preoperative contrast angiography images. **a** An angiography performed via the superior mesenteric artery showing the origin of the replaced right hepatic arterial branches, and the distal pancreatic arterial branches arising from the replaced right hepatic artery. **b** An angiography performed via the celiac artery shows no blood supply from the common hepatic artery to the pancreatic body. The splenic artery is seen to be resected at the root (arrow head) in an earlier surgery. DPA, distal pancreatic artery; RHA, right hepatic artery; SMA, superior mesenteric artery; CHA, common hepatic artery

We planned a post-TG PD that would enable us to preserve the pancreatic body and thereby its functions, in order to prevent the sequelae of postoperative severe malnutrition and disorders of glucose metabolism, and which would also allow us to administer adequate postoperative adjuvant chemotherapy to the patient. However, as the splenic artery had been resected in the earlier procedure, it was necessary to identify the blood vessels perfusing the region including the pancreatic body. A preoperative angiography (AG) was performed, which showed that the dorsal pancreatic artery (DPA) supplying the pancreatic body, had branched from the replaced right hepatic artery (Fig. 3a). Although angiography via both superior mesentic artery (SMA) and celiac artery was performed, the great pancreatic, caudal pancreatic, and the inferior pancreatic arteries could not be identified (Fig. 3b). Based on these results, we concluded that the patient’s DPA was responsible for maintaining the blood flow to the pancreatic body.

**Fig. 4:**
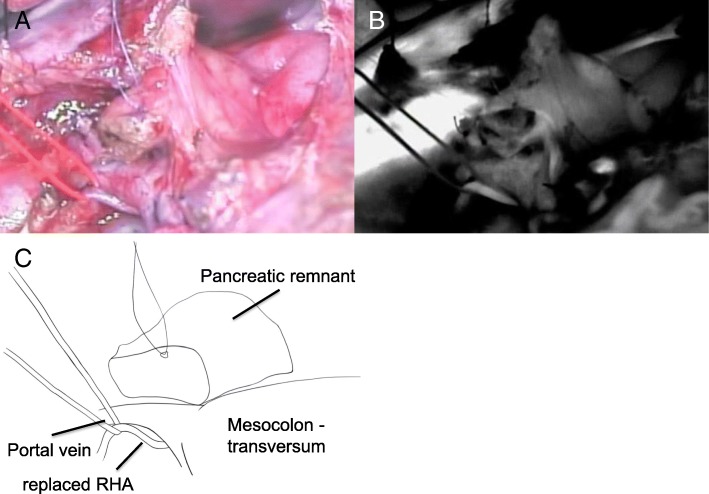
Intraoperative indocyanine green angiography images. The intraoperative indocyanine green angiography images confirming the preservation of the blood flow to the pancreatic remnant. The preserved pancreatic stump is observed to fluoresce clearly. **a** Full color image. **b** Indocyanine green scope image. **c** Schema of the images

**Fig. 5:**
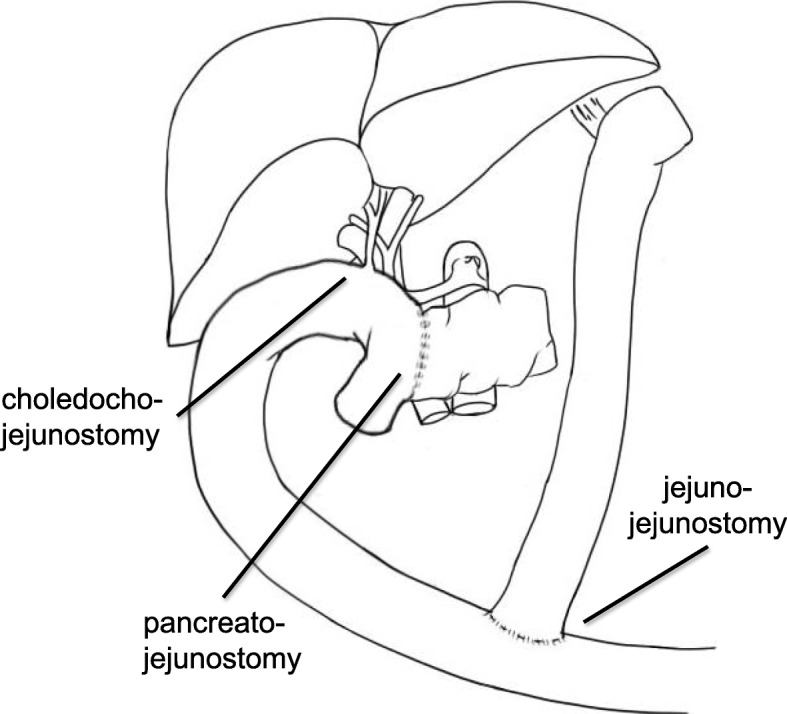
Postoperative abdominal schematic. A postoperative schematic explaining how the earlier esophagojejunostomy was preserved, while performing a reconstruction with a new Roux limb, using the Roux-en-Y method (pancreatojejunostomy, cholangiojejunostomy, and jejunojejunostomy)

**Fig. 6:**
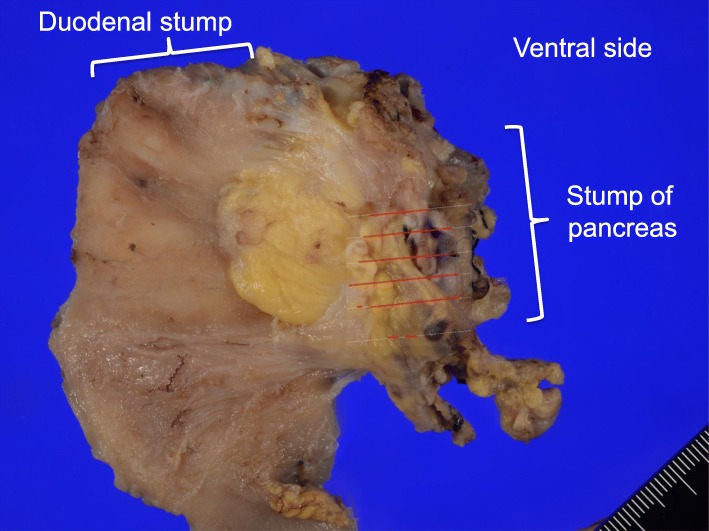
Gross histological examination of the extracted pancreatic tissue. A 19-mm-sized adenocarcinoma is observed localized to the head of the pancreas. Direct invasion of the SMV is seen, but invasion of the arteries and extra-pancreatic nerve plexus is not observed. The red lines indicate the invasive adenocarcinoma region. The margin was tumor-free, including an achievement of R0 resection

A pancreatic body-preserving PD along with an SMV resection was performed. Using intraoperative deep manipulation, the DPA was carefully left intact. In order to prevent injury to the DPA, we only performed partial lymph node dissection around the SMA. We utilized intraoperative ultrasonography to accurately identify the tumor margin and thus delineate the line of resection on the pancreas. The pancreatic parenchyma was preserved to the maximum extent while ensuring that a safe, margin-negative resection was performed. Following the resection, the patient was administered an intravenous injection of 7.5-mg indocyanine green (ICG) to check the blood supply to the pancreatic remnant. The unimpaired blood flow was confirmed when the pancreatic body demonstrated an immediate fluorescence in response (Fig. 4). A subsequent Roux-en-Y reconstruction (pancreatojejunostomy, cholangiojejunostomy, jejunojejunostomy) using a new Roux limb was performed (Fig. 5). The operation was completed in 459 min, during which period, a 250-ml blood loss was recorded. Histopathological examination of the resected specimen showed an R0 resection with safe margins (Fig. 6), no lymph node metastasis, and a moderate effect of the preoperative therapeutic measures. The tumor was staged grade IIa, as per Evan’s histological classification [7], and T3N0M0 and stage IIA according to the 7th edition TNM classification published by the Union for International Cancer Control (UICC) for pancreatic cancer. Furthermore, the pancreatic cancer was diagnosed as a moderately differentiated tubular adenocarcinoma, ly0, v2, ne1, mpd0, and T3N0M0 Stage IIA according to the General Rules for the Study of Pancreatic Cancer (7th edition).

**Fig. 7:**
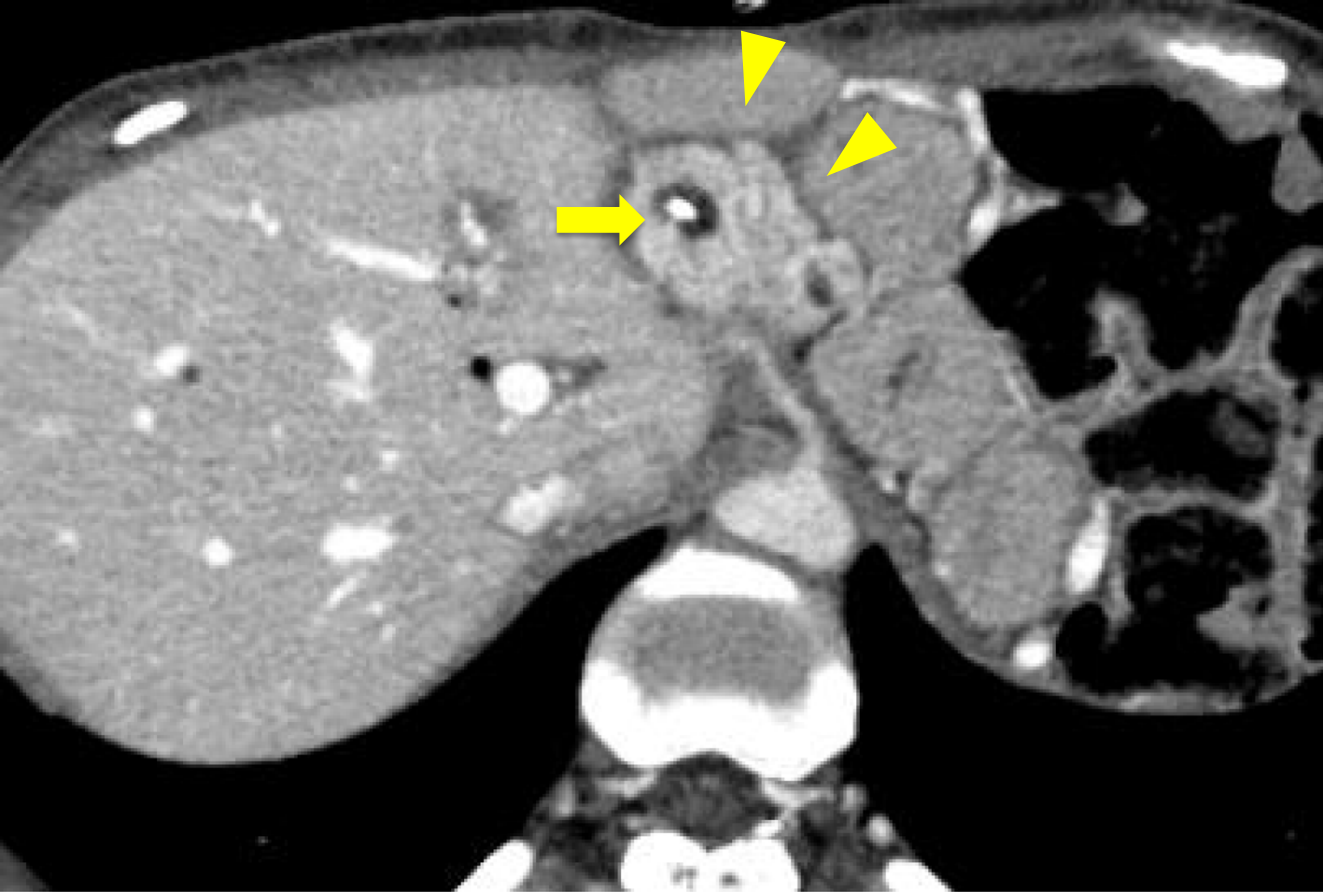
Postoperative abdominal computed tomography scan image. An enhanced abdominal computed tomography scan performed after completing the central pancreatic body-preserving pancreatoduodenectomy showing good blood supply to the pancreatic remnant (arrow head). A pancreatic duct lost-tube is indicated by the arrow

**Fig. 8:**
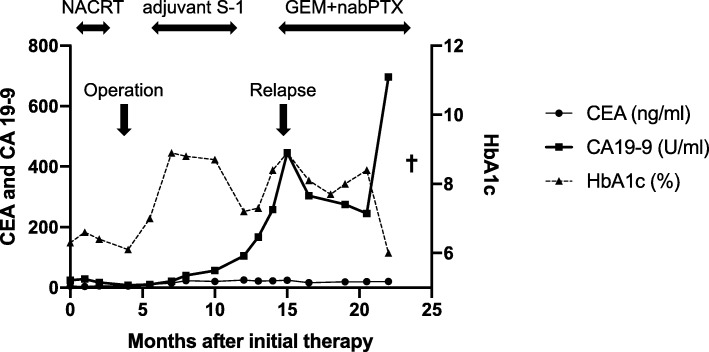
The transition of the tumor markers and HbA1c levels during treatment. A graphical representation of the transition of both carcinoembryonic antigen and CA 19–9 levels along with that of the glycosylated hemoglobin values, indicating a recurrence of peritoneal dissemination 12 months after the surgery, following which, chemotherapy was resumed. CEA, carcinoembryonic antigen; HbA1c, glycosylated hemoglobin; S-1, tegafur, gimeracil, and oteracil potassium; NACRT, neo-adjuvant chemoradiotherapy; GEM+nabPTX, chemotherapy with Gemcitabine and nab-paclitaxel

The course of recovery was uneventful, and the patient was discharged on postoperative day 13. The stable blood supply to the remnant pancreas was confirmed on an enhanced CT scan (Fig. 7). The patient had required insulin therapy for a short duration postoperatively but eventually was able to achieve good glycemic control using an oral hypoglycemic agent. At discharge, the patient weighed 35.2 kg (body mass index, 15.4 kg/m^2^) and had lost 1.7 kg, as compared to her preoperative weight. Adjuvant chemotherapy with S-1 was then administered for 6 months. The transition in the levels of tumor markers and HbA1c values during the course of treatment have been graphically represented in Fig. 8. The patient passed away 21 months after the operation and 24 months after the start of preoperative therapy, due to a recurrence of peritoneal metastasis.

## Discussion

A total pancreatectomy after a TG can markedly lower the patient’s quality of life by causing severe nutritional disorders. In the present case, we were able to safely preserve the pancreatic body by employing the techniques of preoperative AG and intraoperative ICG fluorescence examinations.

It is necessary to perform a detailed diagnostic imaging of the vessels around the pancreas before a pancreatic surgery, as anatomical variations often exist. The DPA is reported to originate from the splenic artery in 40%, the common hepatic artery (CHA) in 25.7%, the SMA in 20%, the celiac artery in 8.6%, and the jejunal artery and middle colic artery in 5.7% of all cases [8]. Recent advances in CT technology have enabled a more detailed assessment of vascular pathways. Nevertheless, an accurate delineation of the pathways of fine arteries like the DPA remains difficult. In our case, we were fortunately able to identify the DPA entirely, using preoperative AG, and further used intraoperative ICG florescence to observe the perfusion of the pancreatic body in real time, following the resection of the pancreatic head. The ICG fluorescence technique is often used to evaluate organ blood flow in various procedures, e.g., a gastric tube reconstruction in esophageal surgery [9]. There have been few reports of its utilization in conserving pancreatic parenchyma, but we could employ the technique successfully in our patient, thus demonstrating its usefulness.

It was difficult to evaluate the patient’s endocrine and exocrine functions after the operation, in this case. However, the patient did not require insulin therapy postoperatively, which indicated that some level of pancreatic function was preserved. Eschewing a total pancreatectomy allowed the surgeon to let the patient maintain her quality of life. We could significantly reduce the severity of the disorders of nutritional and glucose metabolism, which would have been a grim consequence of a total PD. This helped suppress further weight-loss and enabled the patient to complete the course of postoperative adjuvant therapy. According to recent reports, the median survival time of borderline resectable pancreatic cancer patients, who have undergone preoperative treatment, is 21–40 months [10–15]. The 24-month survival period, as measured from the start of initial therapy, indicated a good balance of the combined therapeutic effects of the operative procedure and the postoperative adjuvant therapy.

## Conclusions

All operative procedures for pancreatic cancer should be performed with an aim to preserve its function, to allow the patient to maintain an optimum quality of life. Due consideration should be given to the administration of postoperative adjuvant therapy, while still attempting to ensure a complete cure. Further, the techniques of preoperative AG and intraoperative ICG fluorescence can be utilized to accurately evaluate the pancreatic vascular supply and therefore aid the operating surgeon in preserving functional pancreatic parenchyma to the maximum extent, while achieving a safe, margin-negative resection.

## Abbreviations

AG: Angiography
CHA: Common hepatic artery
CT: Computed tomography
DP: Distal pancreatectomy
DPA: Dorsal pancreatic artery
ICG: Indocyanine green
NCCN: National Comprehensive Cancer Network
PD: Pancreatoduodenectomy
S-1: Tegafur, gimeracil, and oteracil potassium
SMA: Superior mesenteric artery
SMV: Superior mesenteric vein
TG: Total gastrectomy
UICC: Union for International Cancer Control
